# Supplementary material for: In vivo simultaneous nonlinear absorption Raman and fluorescence (SNARF) imaging of mouse brain cortical structures
Source: Commun Biol. 2022 Mar 10;5:222. doi: 10.1038/s42003-022-03166-6 (PMC8913696; doi:10.1038/s42003-022-03166-6)
Supplement: Supplementary file 2 — Supplementary Materials [file 42003_2022_3166_MOESM2_ESM.pdf]

Title:

In Vivo Simultaneous Nonlinear Absorption Raman and Fluorescence (SNARF) Imaging of Mouse Brain Cortical Structures

Authors:

Andrew T. Francis[1], Bryce Manifold[1], Elena C. Carlson[1], Ruoqian Hu[1], Andrew H. Hill[1], Shuaiqian Men[1], Dan Fu[1]*

Affiliations:

1. Department of Chemistry, University of Washington, Seattle, Washington 98195, United States

* Corresponding author: danfu@uw.edu

This PDF file includes:

Figs S1 – S6

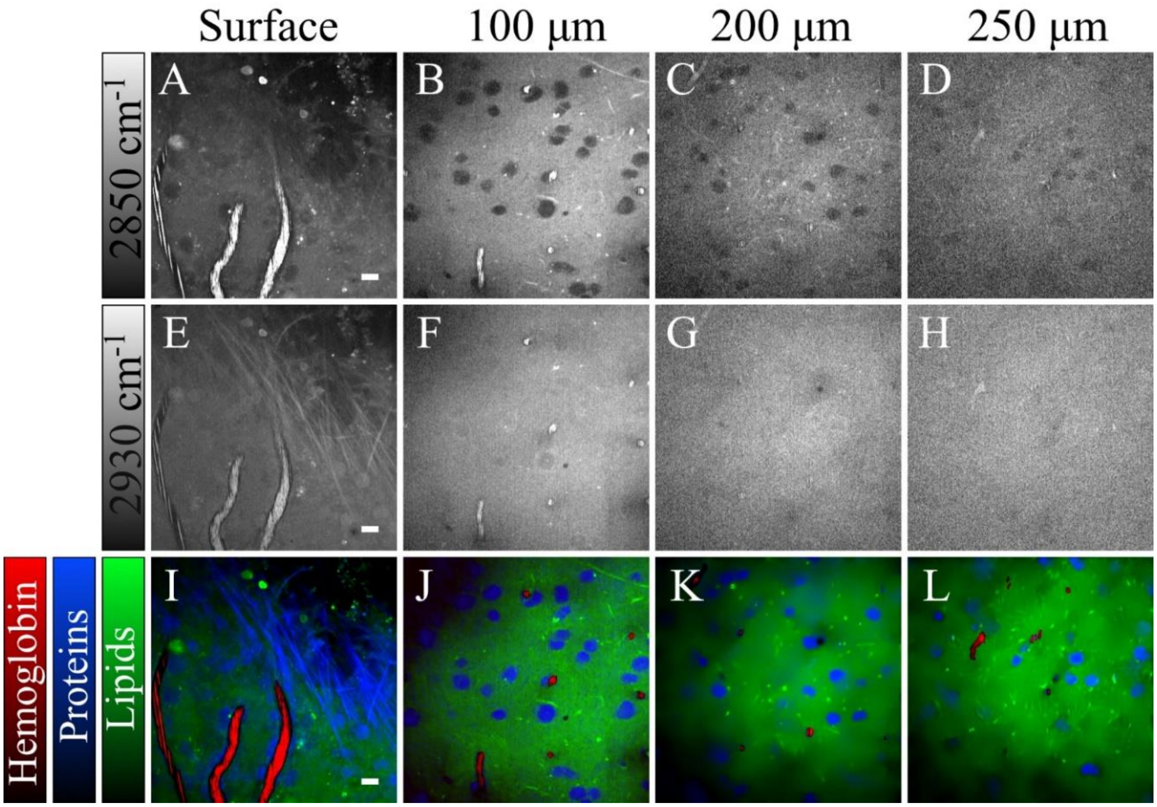

Figure S1. Representative unprocessed (A-H) and processed (I-L) images of *in vivo* SNARF microscopy at the surface (A, E, I) and 100 µm (B, F, J), 200 µm (C, G, K), and 250 µm (D, H, L) below the pial surface. Processing involved deep-learning denoising and spectral separation of SRS and TAM. Scale bar: 10 µm.

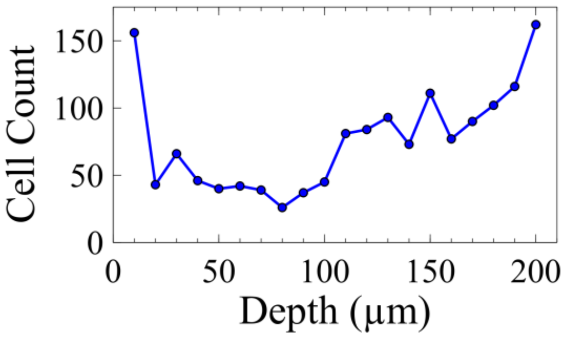

Figure S2. Depth dependent cell count for a P246 mouse. The increase in cells near 100 μm represents the transition from layer 1 to layer 2/3 of the cortex.

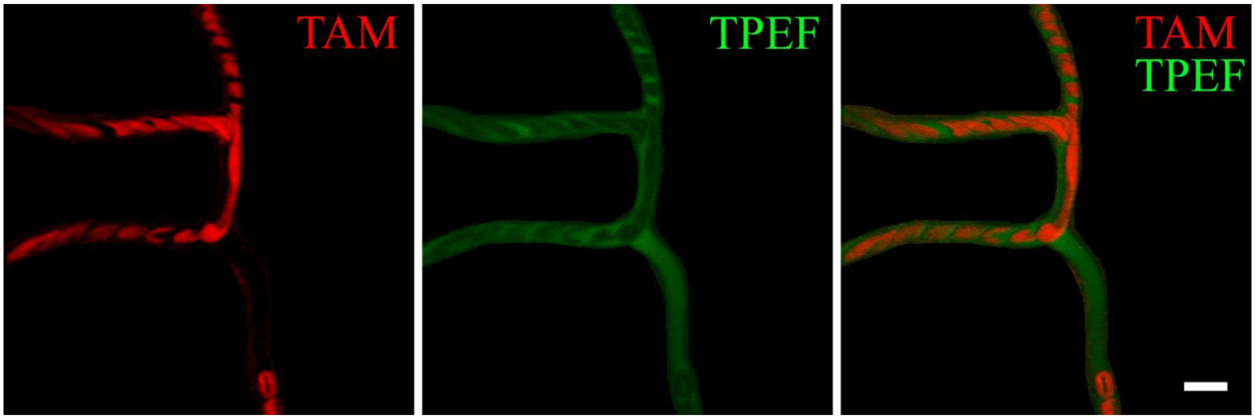

Figure S3. In vivo imaging of a mouse brain capillary using the intrinsic optical absorption of hemoglobin (red). The capillary is also stained with FITC (green) to demonstrate the orthogonality of the two techniques. Scale bar: 10 µm.

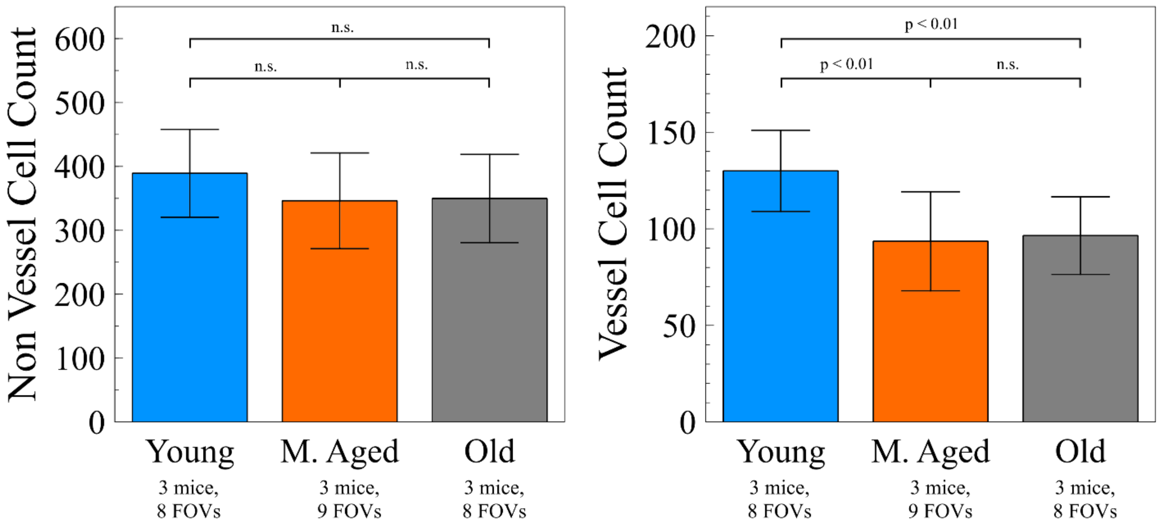

Figure S4. Cell populations in the top 200 µm for three groups of mice: young (P37-P48), middle-aged (P209-P246), and old(P630-P631). Cells were stratified based on distance from neighboring blood vessels. Error bars represent standard deviation n.s.: not significant.

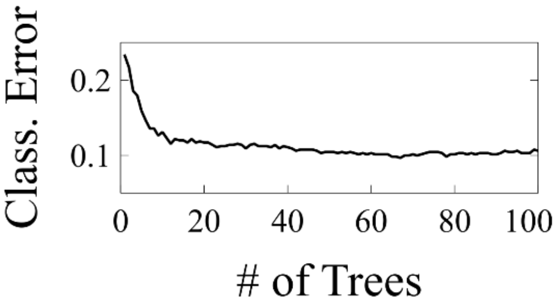

Figure S5. Random forest prediction error as number of trees increases. Classifier was built on a 70/30 separation of train/test for 661 endothelial cells, 134 pericytes, and 120 unlabeled cells.

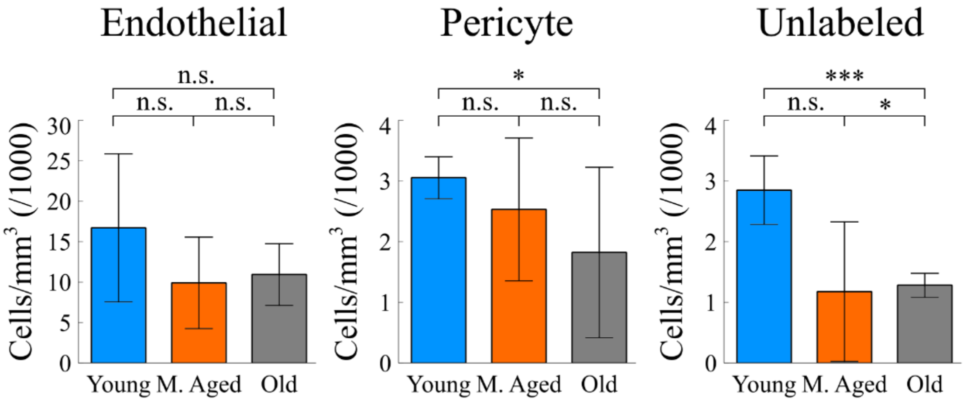

Figure S6. Age-dependent capillary-lining cell population density in layer 1 of mouse brain cortex. Three cell types were identified based on machine learning: endothelial cells, pericytes, and unlabeled cells. Three age groups were measured: young (P37-P92, n=5), middle-aged (P209-P246, n=3), and old (P630-P631, n=3). The statistical significances between age groups are provided as not significant (n.s.), $p<0.1$ (*), or $p<0.005$ (**). Error Bars represent standard
