# Supplementary material for: In vivo simultaneous nonlinear absorption Raman and fluorescence (SNARF) imaging of mouse brain cortical structures
Source: Commun Biol. 2022 Mar 10;5:222. doi: 10.1038/s42003-022-03166-6 (PMC8913696; doi:10.1038/s42003-022-03166-6)
Supplement: Supplementary file 3 — Description of Additional Supplementary Files [file 42003_2022_3166_MOESM3_ESM.pdf]

Description of Additional Supplementary Files

File name: Supplementary Data 1

Description: Raw data for Figures 1h, 2e, 2f, 2h, 2i, 3h, 3i, and 3j.
